# Supplementary material for: Global and transcription-coupled repair of 8-oxoG is initiated by nucleotide excision repair proteins
Source: Nat Commun. 2022 Feb 21;13:974. doi: 10.1038/s41467-022-28642-9 (PMC8861037; doi:10.1038/s41467-022-28642-9)
Supplement: Supplementary file 1 — Supplementary Information [file 41467_2022_28642_MOESM1_ESM.pdf]

Supplementary Figures:

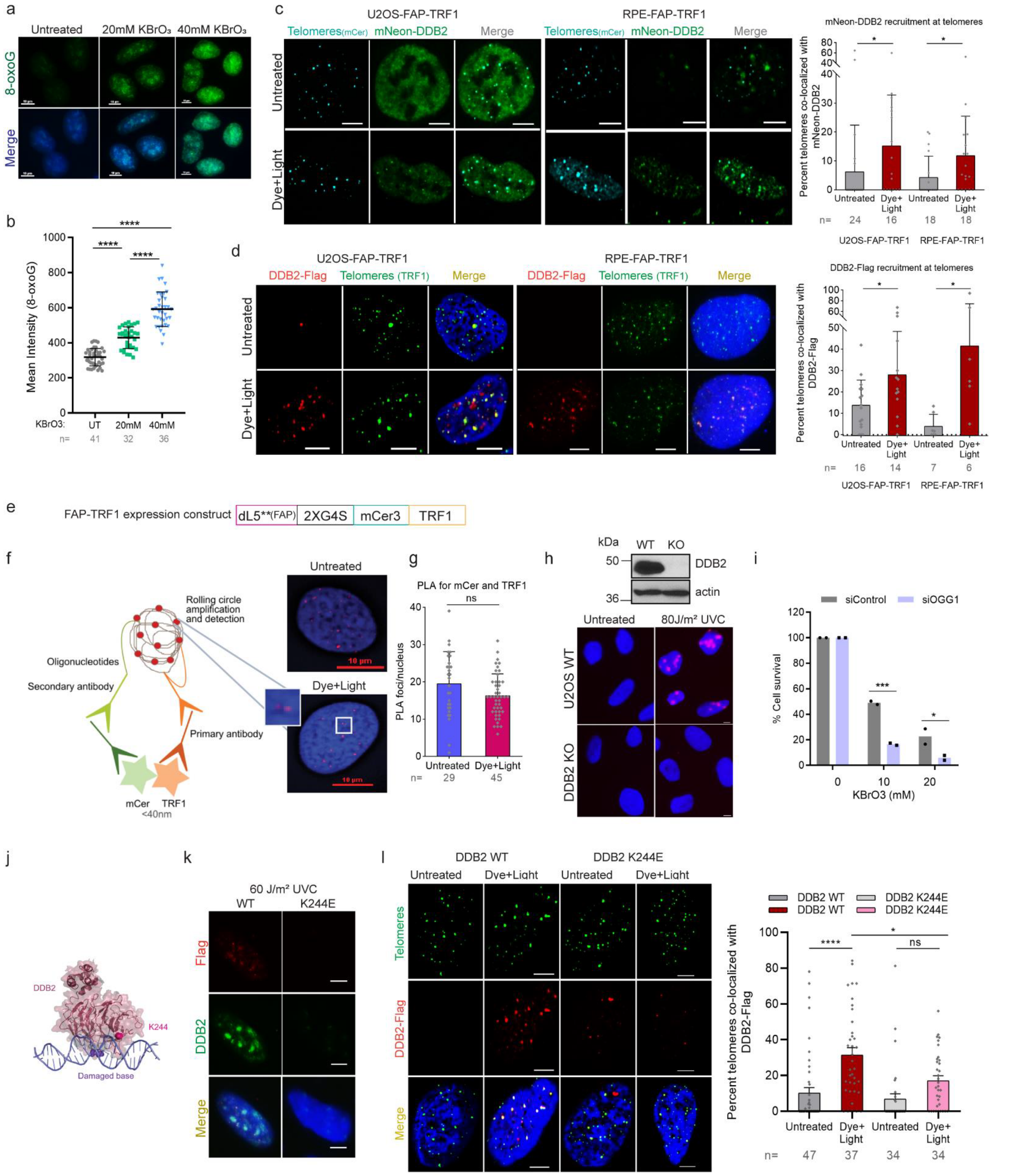

Supplementary Figure 1 (Related to Figure 1)**: Robust recruitment of WT DDB2, but not XP-E K244E variant, to telomeric 8-oxoG: a, b** Immunofluorescence for 8-oxoG after treating cells with $KBrO_3$ at indicated doses, for an hour. **c** Recruitment of mNeon-DDB2 at telomeres after dye (100 nM, 15 min) plus light (660 nm, 10 min) treatment in U2OS and RPE-hTERT cells. mCerulean and mNeon fluorescence was directly observed under the microscope. **d** Recruitment of DDB2-Flag at telomeres after dye (100 nM, 15 min) plus light (660 nm, 10 min) treatment in U2OS and RPE-hTERT cells. **e** Schematic of the FAP-TRF1 overexpression construct stably expressed in U2OS and RPE-hTERT cells. **f** Schematic representation of proximity ligation assay (PLA). **g** Antibodies against mCerulean (mCer3) and TRF1 were used as a positive control to validate the PLA conditions. **h** Western blot and Immunofluorescence (after local UVC exposure through 2 µm PC membrane) for DDB2 in U2OS-FAP-TRF1 wildtype (WT) and DDB2 knockout (KO) cells. **i** Clonogenic cell survival curves in U2OS cells transfected with control or OGG1 siRNA and treated with a range of concentrations of $KBrO_3$. Data represents mean ± SEM from two independent experiments, each with three technical replicates. **j** Structure of DDB2 (dark red) bound to damaged (Purple) duplex DNA. Lysine 244 (pink) was mutated to glutamic acid (K244E). **k** Immunofluorescence for DDB2-Flag (after local UVC exposure through 2µm PC membrane) in U2OS-FAP-TRF1 cells overexpressing WT or K244E variant of DDB2-Flag. **l** Recruitment of DDB2-WT and DDB2-K244E to 8-oxoG sites at telomeres after dye plus light treatment (left). Percentage telomeres colocalized with DDB2-Flag (right). Data represents mean ± SD (b, c, d, g, l) and 'n' represents the number of cells scored for each condition. Data represents mean ± SEM from two independent experiments, performed in triplicate (i). Two-sided Mann Whitney test (c, d), Student's two-tailed t-test (g), one-way ANOVA (Sidak multiple comparison test) (a, l) and two-way ANOVA (Sidak multiple comparison test) (i) was performed for statistical analysis: *p<0.05, **p<0.01, ***p<0.001, ****p<0.0001. Scale: 10 µm (a, f), 5 µm (c, d, h, k, l). Source data are provided as a Source Data file.

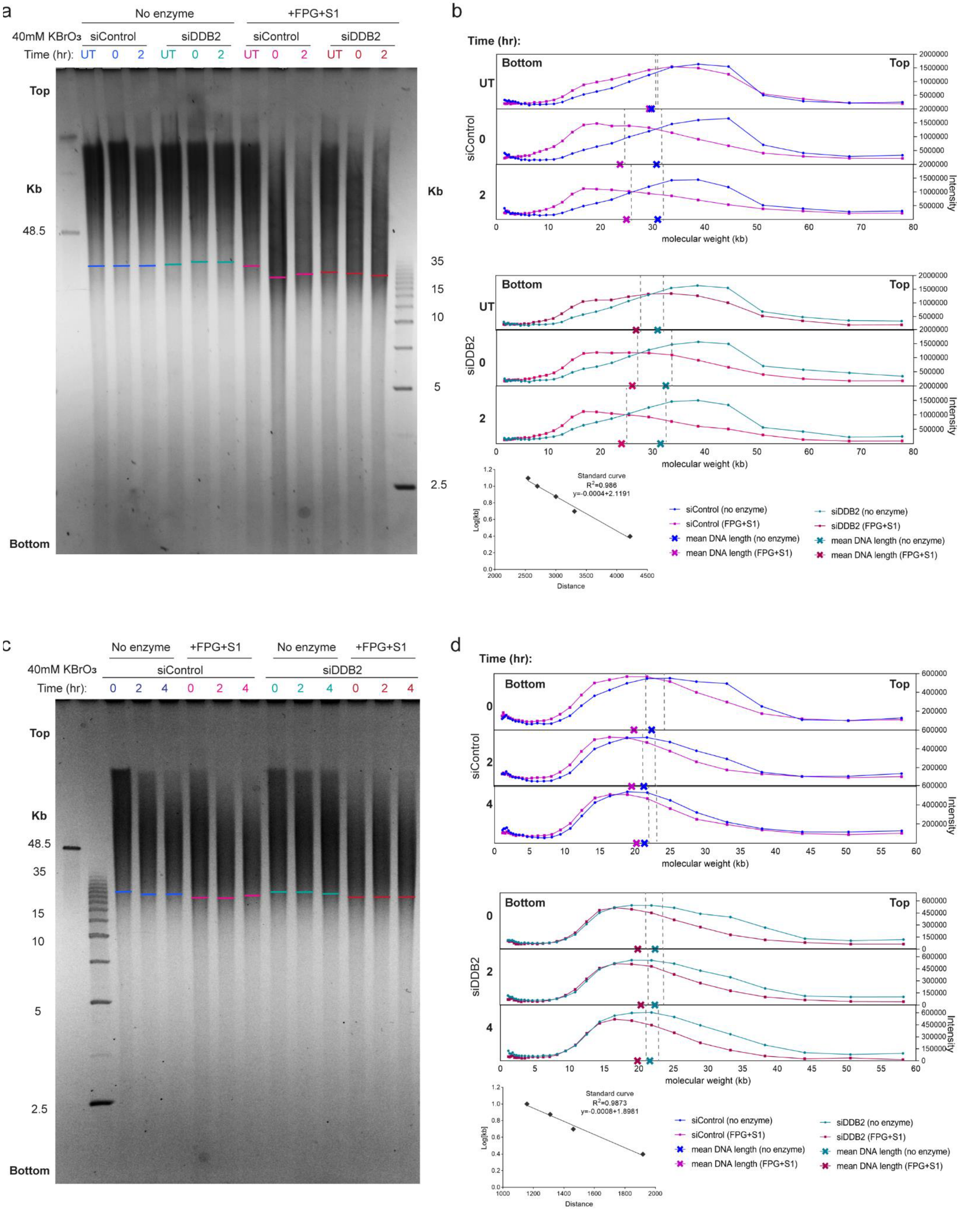
a
No enzyme
+FPG+S1
40mM KBrO3
siControl
siDDB2
siControl
siDDB2
Time (hr): UT 0 2 UT 0 2 UT 0 2 UT 0 2
Top
Kb
48.5
Kb
35
15
10
5
2.5
Bottom
b
Time (hr):
Bottom
Top
UT
0
2
siControl
siDDB2
Intensity
molecular weight (kb)
Standard curve
R2=0.986
y=-0.0004+2.1191
Log[kb]
Distance
siControl (no enzyme)
siControl (FPG+S1)
mean DNA length (no enzyme)
mean DNA length (FPG+S1)
siDDB2 (no enzyme)
siDDB2 (FPG+S1)
mean DNA length (no enzyme)
mean DNA length (FPG+S1)
c
No enzyme
+FPG+S1
No enzyme
+FPG+S1
40mM KBrO3
siControl
siDDB2
Time (hr): 0 2 4 0 2 4 0 2 4 0 2 4
Top
Kb
48.5
35
15
10
5
2.5
Bottom
d
Time (hr):
Bottom
Top
0
2
4
siControl
siDDB2
Intensity
molecular weight (kb)
Standard curve
R2=0.9873
y=-0.0008+1.8981
Log[kb]
Distance
siControl (no enzyme)
siControl (FPG+S1)
mean DNA length (no enzyme)
mean DNA length (FPG+S1)
siDDB2 (no enzyme)
siDDB2 (FPG+S1)
mean DNA length (no enzyme)
mean DNA length (FPG+S1)

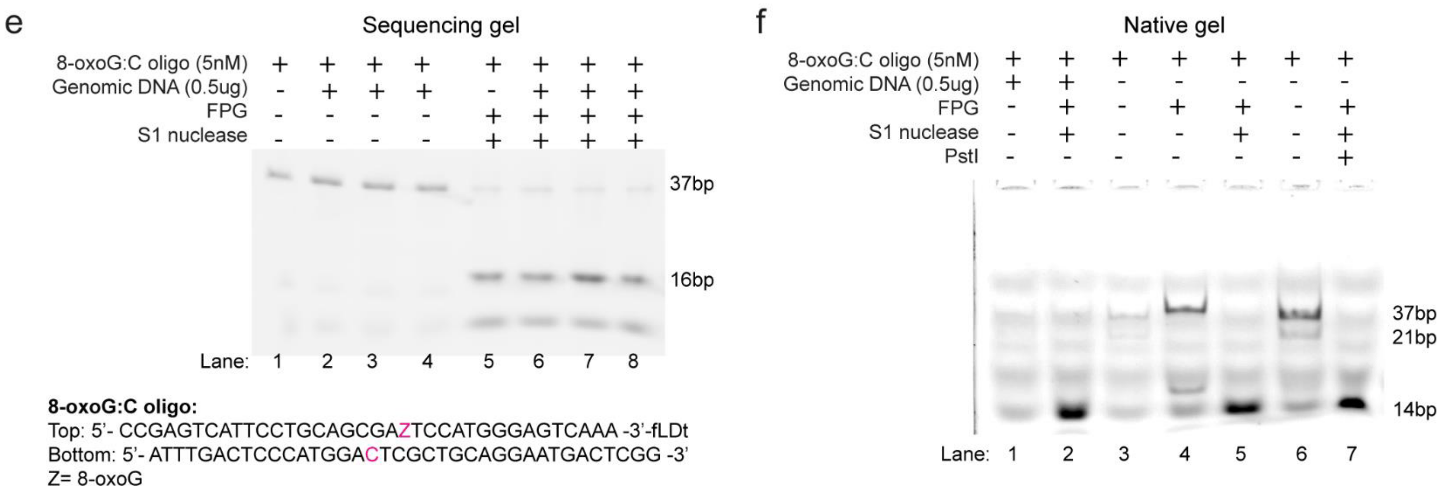

Supplementary Figure 2 (Related to Figure 1): **Repair of 8-oxoG is slower in the absence of DDB2. a** Representative pulse field gel with DNA extracted from cells treated with $KBrO_3$ and harvested immediately or allowed to recover for 2 hours. Lanes show DNA migration in cells transfected with control or DDB2 siRNA and either with no enzyme or treated with FPG+S1 to generate DSBs. DNA containing DSBs migrate faster on the gel. Colored lines indicate the weighted mean of DNA length. **b** Plots of band intensity against molecular weight (kb). Cross (x) and dashed line represent the weighted mean DNA length for each condition. Top grid consisting of the well was excluded from the plot for clarity. Standard curve used to calculate weighted mean DNA length (bottom). **c** Representative pulse field gel with DNA extracted from cells treated with $KBrO_3$ and harvested immediately or allowed to recover for 2 or 4 hours. Lanes show DNA migration in cells transfected with control or DDB2 siRNA and either with no enzyme or treated with FPG+S1 to generate DSBs. DNA containing DSBs migrate faster on the gel. Colored lines indicate the weighted mean of DNA length. **d** Plots of band intensity against molecular weight (kb). Cross (x) and dashed line represent the weighted mean DNA length for each condition. Top grid consisting of the well was excluded from the plot for clarity. Standard curve used to calculate weighted mean DNA length (bottom). Source data are provided as a Source Data file. **e, f** Control experiments using 0.5µg genomic DNA and a 100-fold molar excess of labelled 37-mer oligo containing 8-oxoG treated with FPG and S1 nuclease. Samples were run on a 10% sequencing gel (compare lane 5 and 6-8) and a native gel (compare lane 2 and 5) to confirm that all the oligo was incised in the presence of genomic DNA.

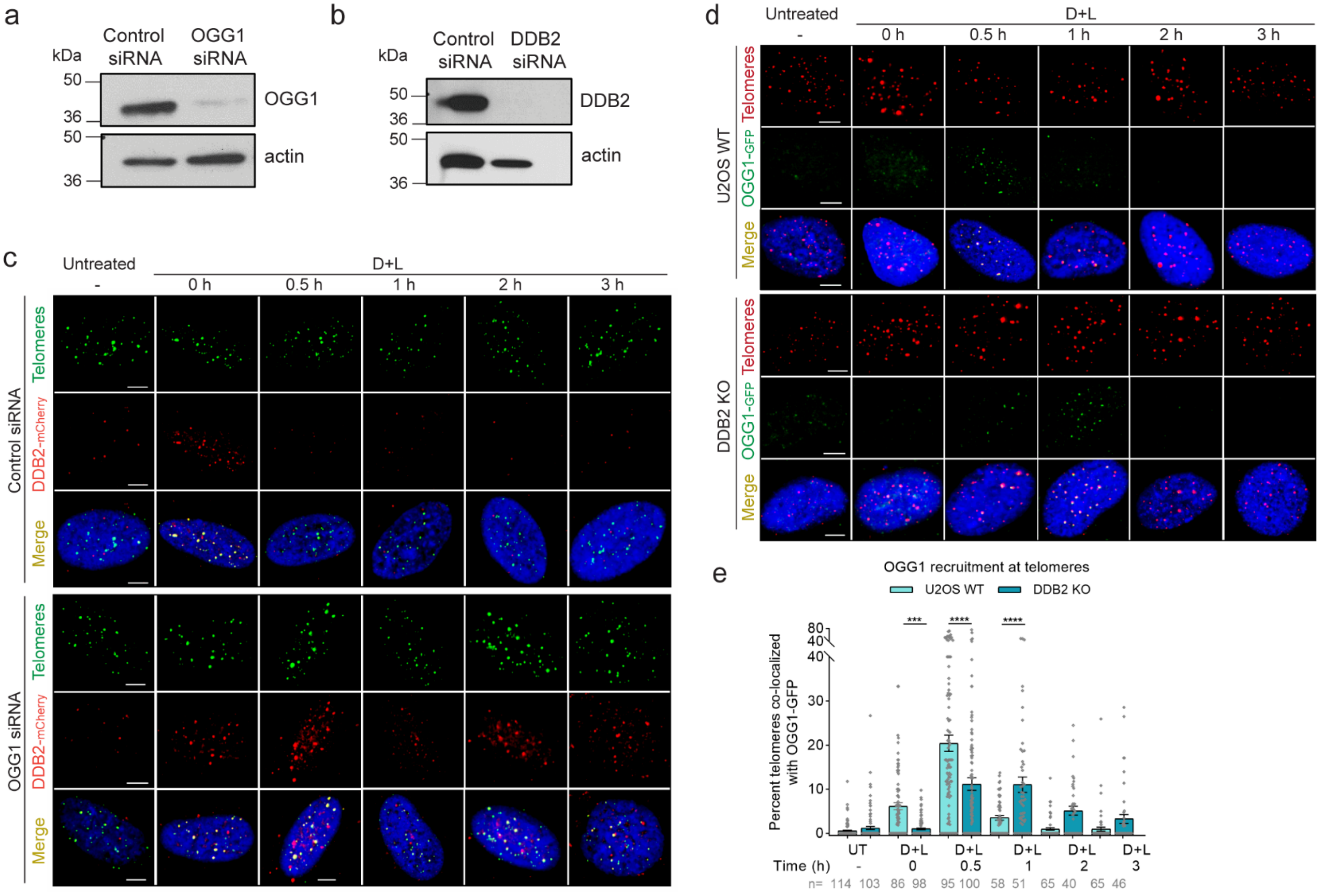

Supplementary Figure 3 (Related to Figure 2)**: DDB2 is required for efficient OGG1 recruitment to 8-oxoG: a** Western blot for OGG1 expression in U2OS-FAP-TRF1 cells transfected with control or OGG1 siRNA, 48 hours post siRNA transfection. **b** Western blot for DDB2 expression in U2OS-FAP-TRF1 cells transfected with control or DDB2 siRNA, 48 hours post siRNA transfection. **c** Recruitment of DDB2-mCherry to damaged telomeres after dye (100 nM, 15 min) plus light (660 nm, 10 min) treatment in U2OS-FAP-TRF1 transfected with control or OGG1 siRNA. **d** Recruitment of OGG1-GFP to damaged telomeres after dye plus light treatment in U2OS WT and DDB2 KO cells. **e** Quantification of d. Data represents mean ± SEM from two independent experiments. ‘n’ represents the number of cells scored for each condition. One-way ANOVA (Sidak multiple comparison test): *p<0.05, **p<0.01, ****p<0.0001. Scale: 5 µm. Source data are provided as a Source Data file.

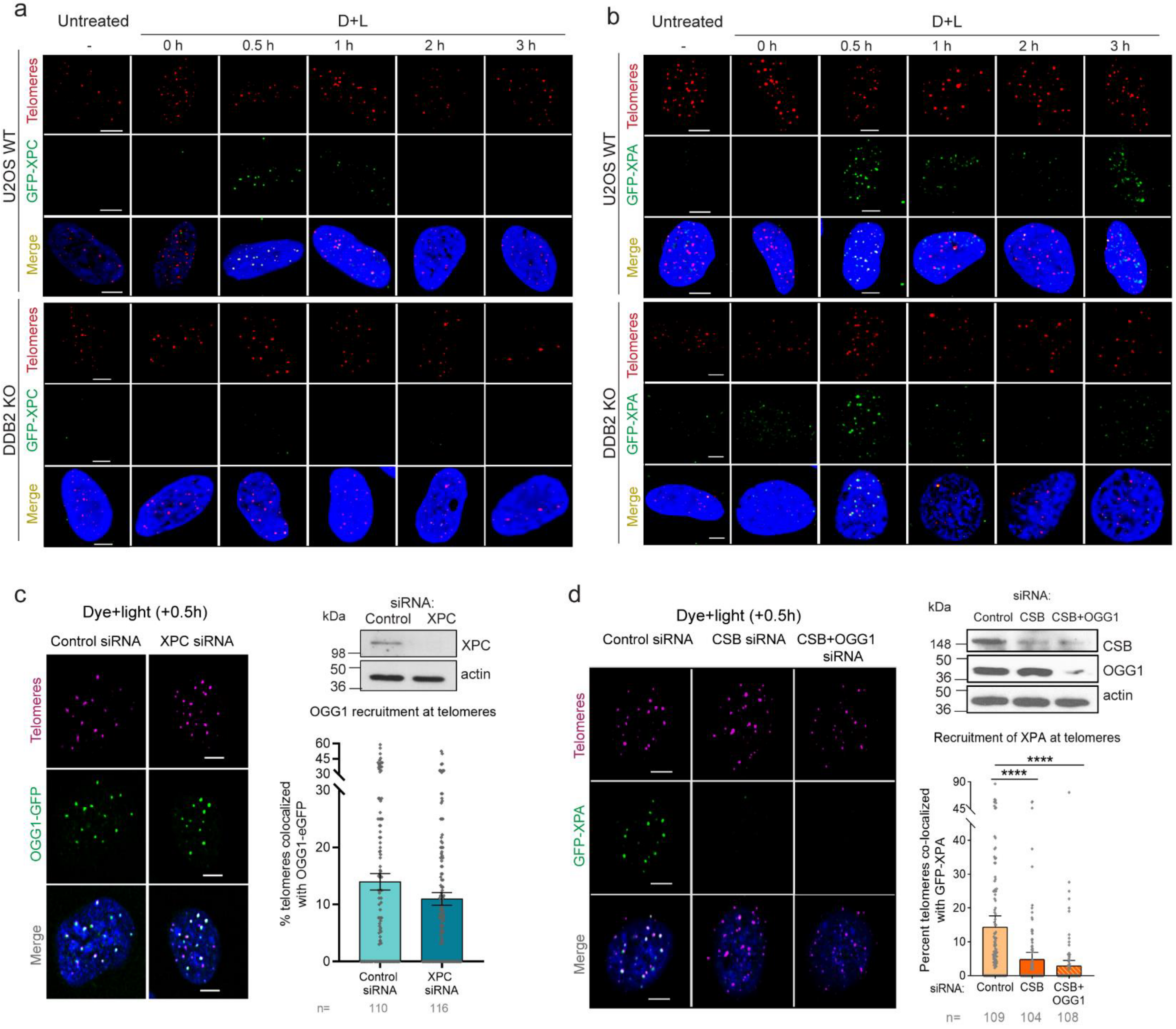

Supplementary Figure 4 (Related to Figure 3)**: DDB2 recruits XPC to telomeric 8-oxoG, while XPA recruitment is transcription-coupled and independent of DDB2: a, b** Representative images showing recruitment of GFP-XPC (A) or GFP-XPA (B) to 8-oxoG sites at telomeres after dye (100 nM, 15 min) plus light (660 nm, 10 min) treatment in U2OS WT and DDB2 KO cells, over 3 hours. **c** Recruitment of OGG1-GFP to telomeric 8-oxoG in cells transfected with control or XPC siRNA. Western blot shows XPC protein levels, 48 hours post siRNA transfection. **d** Accumulation of GFP-XPA at damaged telomeres in cells transfected with control, CSB or CSB+OGG1 siRNA. Western blot shows respective protein expression, 48 hours post siRNA transfection. Data (c, d) represents mean ± SEM from two independent experiments. 'n' represents the number of cells scored for each condition. One-way ANOVA (Sidak multiple comparison test): ****$p<0.0001$. Scale: 5µm. Source data are provided as a Source Data file.

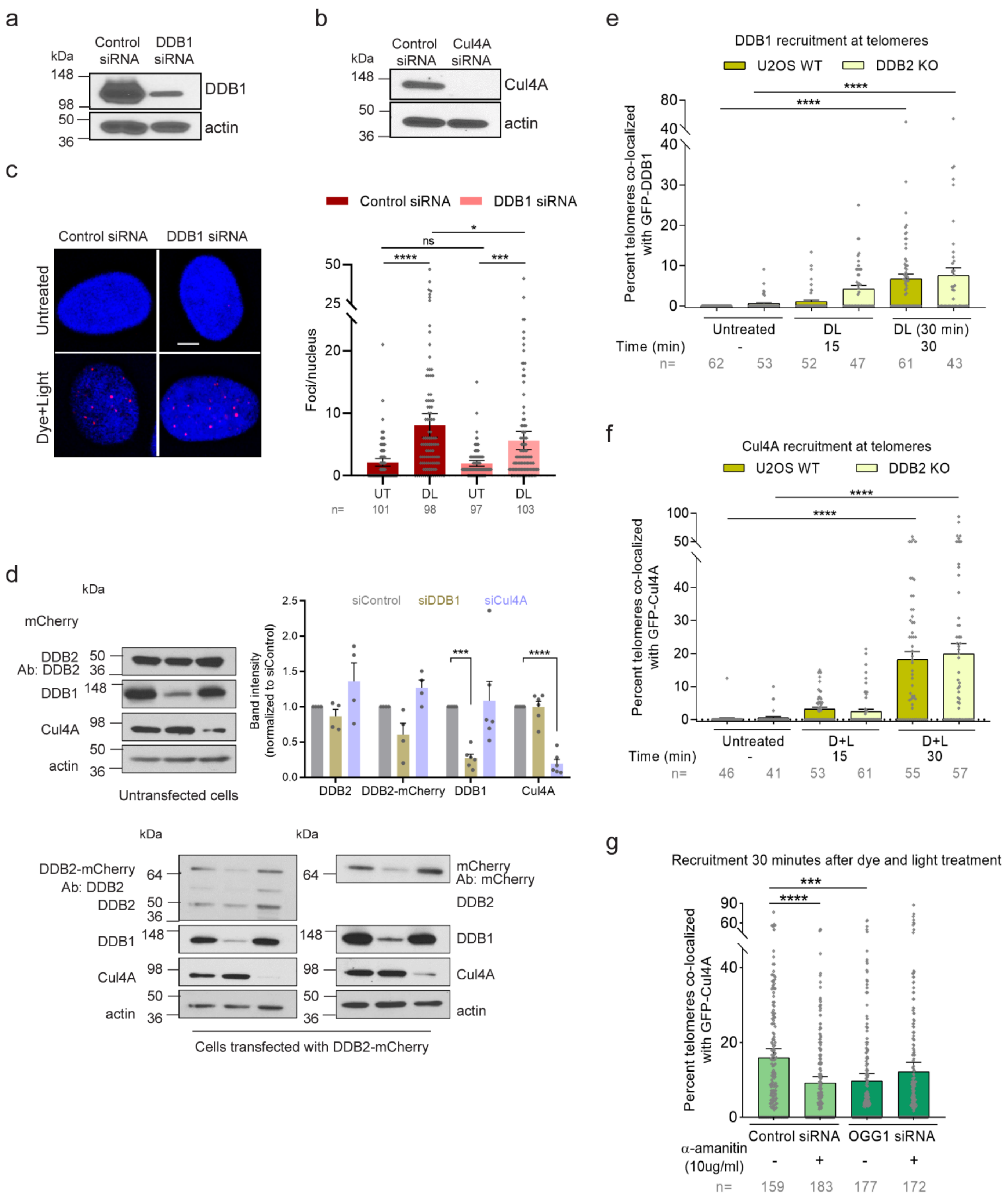
a
kDa
Control siRNA
DDB1 siRNA
148
98
50
36
DDB1
actin
b
Control siRNA
Cul4A siRNA
Cul4A
actin
c
Control siRNA
DDB1 siRNA
Untreated
Dye+Light
Foci/nucleus
UT
DL
n=
101
98
97
103
ns
d
mCherry
DDB2
Ab: DDB2
DDB1
Cul4A
actin
Untransfected cells
siControl
siDDB1
siCul4A
Band intensity
(normalized to siControl)
DDB2
DDB2-mCherry
DDB2-mCherry
Ab: DDB2
mCherry
Ab: mCherry
Cells transfected with DDB2-mCherry
e
DDB1 recruitment at telomeres
U2OS WT
DDB2 KO
Percent telomeres co-localized
with GFP-DDB1
Untreated
DL
DL (30 min)
Time (min)
f
Cul4A recruitment at telomeres
Percent telomeres co-localized
with GFP-Cul4A
D+L
g
Recruitment 30 minutes after dye and light treatment
Control siRNA
OGG1 siRNA
α-amanitin
(10ug/ml)
159
183
177
172

Supplementary Figure 5 (Related to Figure 4)**: DDB2 binds sparse telomeric 8-oxoG independently of the DDB1-Cul4A-RBX1 E3 ligase: a** Western blot for DDB1 and **b** Cul4A in U2OS-FAP-TRF1 cells treated with DDB1 or Cul4A siRNA. **c** PLA for DDB2-mCherry and TRF1 in cells transfected with control or DDB1 siRNA. **d** Western blot and quantification of endogenous and overexpressed DDB2 protein levels in cells transfected with control, DDB1 or Cul4A siRNA. Antibodies (Ab) used for DDB2 are indicated in the figure. **e** GFP-DDB1 and **f** GFP-Cul4A recruitment to telomeric 8-oxoG after dye (100 nM, 15 min) plus light (660 nm, 10 min) treatment in WT and DDB2 KO cells. **g** Colocalization of GFP-Cul4A at damaged telomeres in U2OS-FAP-TRF1 cells transfected with control or OGG1 siRNA and pre-treated with transcription inhibitor α-amanitin. Data represents mean ± SEM from one (e, f), two (c, d) or three (g) independent experiments. 'n' represents the number of cells scored for each condition. One-way ANOVA (Sidak multiple comparison test) (c, e, f, g) and two-way ANOVA (Sidak multiple comparison test) (d) was performed for statistical analysis: ns: not significant, *p<0.05, **p<0.01, ***p<0.001, ****p<0.0001. Scale: 5μm. Source data are provided as a Source Data file.

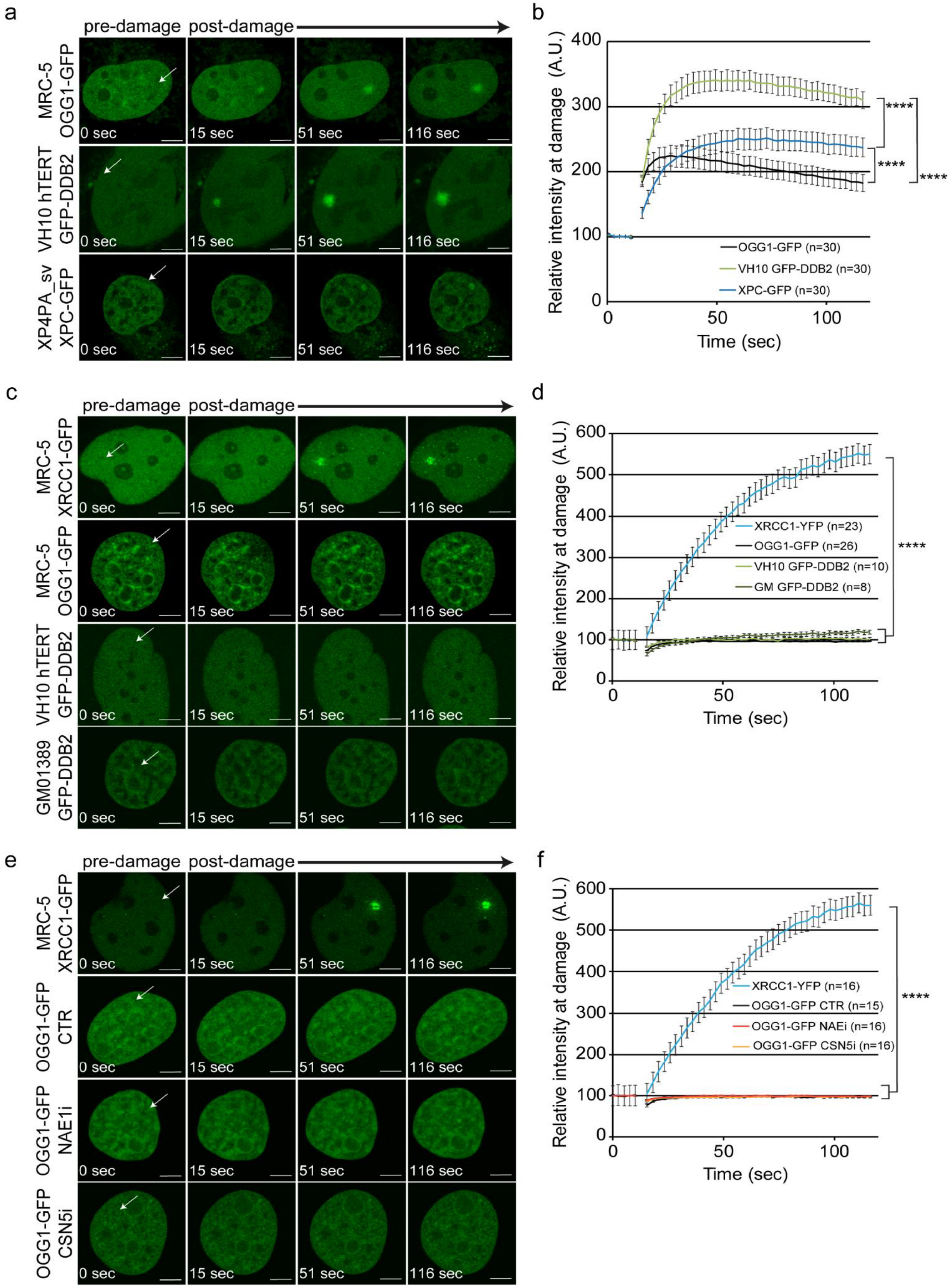
a
pre-damage
post-damage
MRC-5 OGG1-GFP
VH10 hTERT GFP-DDB2
XP4PA_sv XPC-GFP
0 sec
15 sec
51 sec
116 sec
b
Relative intensity at damage (A.U.)
Time (sec)
OGG1-GFP (n=30)
VH10 GFP-DDB2 (n=30)
XPC-GFP (n=30)
****
c
MRC-5 XRCC1-GFP
MRC-5 OGG1-GFP
VH10 hTERT GFP-DDB2
GM01389 GFP-DDB2
d
XRCC1-YFP (n=23)
OGG1-GFP (n=26)
VH10 GFP-DDB2 (n=10)
GM GFP-DDB2 (n=8)
e
OGG1-GFP CTR
OGG1-GFP NAE1i
OGG1-GFP CSN5i
f
XRCC1-YFP (n=16)
OGG1-GFP CTR (n=15)
OGG1-GFP NAEi (n=16)
OGG1-GFP CSN5i (n=16)

Supplementary Figure 6 (Related to Figure 5)**: OGG1-GFP and GFP-DDB2 do not accumulate at SSBs: a** Representative time-lapse picture of GFP-DDB2, OGG1-GFP and XPC-GFP accumulation at micro-irradiated (405 nm laser) sub-nuclear area, indicated by arrows, in the presence of 50μM Ro 19-8022 photosensitizer. **b** Quantification of accumulation kinetics of GFP-DDB2, OGG1-GFP and XPC-GFP (as shown in a). **c** Representative time-lapse pictures of XRCC1-YFP, OGG1-GFP and GFP-DDB2 accumulation at high power micro-irradiated (405 nm laser) sub-nuclear area in the absence of the photosensitizer, indicated by arrows. **d** Quantification of accumulation kinetics of XRCC1-YFP, OGG1-GFP and GFP-DDB2 (as shown in c). **e** Representative time-lapse pictures of XRCC1-YFP and OGG1-GFP accumulation at high power micro-irradiated (405 nm laser) sub-nuclear area in the absence of the photosensitizer, indicated by arrows. Cells were pre-treated with DMSO (CTR), neddylation inhibitor (NEDDi) or de-neddylation inhibitor (deNEDDi) for 1.5 hours. **f** Quantification of accumulation kinetics of XRCC1-YFP and OGG1-GFP (as shown in E). Scale bars: 5 µm. Data were normalized to the background and represent mean ± SEM from three independent experiments. Total number of cells ‘n’ measured are indicated in figure legends. ****P < 0.001, analyzed by ROC curve analysis. Source data are provided as a Source Data file.

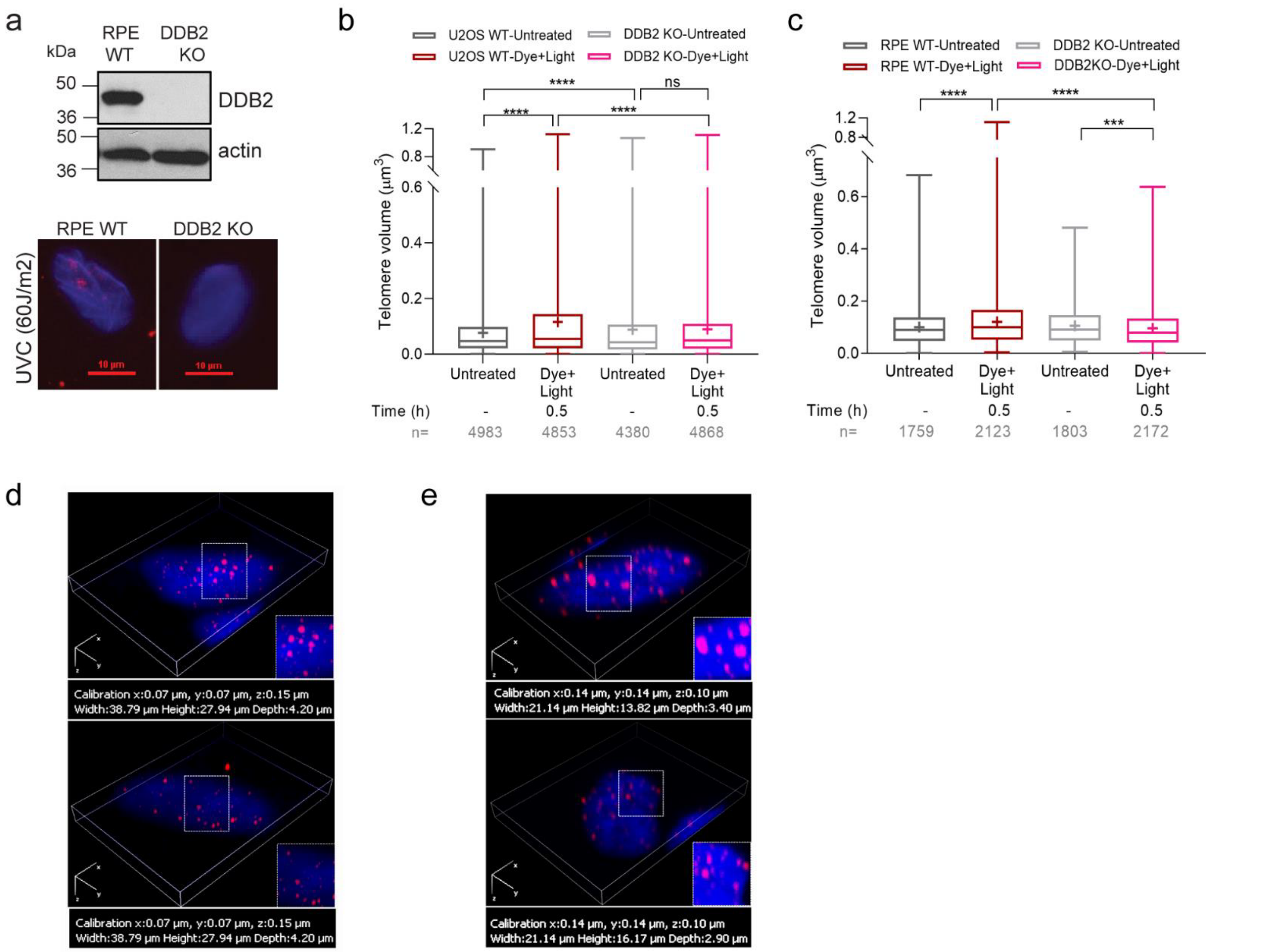

Supplementary Figure 7 (Related to Figure 6): **DDB2 mediates chromatin decompaction at sites of telomeric 8-oxoG: a** Western blot and immunofluorescence for DDB2 in RPE-FAP-TRF1 WT and DDB2 KO cells. **b, c** Telomere volumes were measured in untreated, and cells treated with dye (100 nM, 15 min) plus light (660 nm, 10 min) in WT and DDB2 KO cells. Cells were fixed 30 minutes post treatment. **b** U2OS-FAP-TRF1 and **c** RPE-FAP-TRF1 cells. Center line indicates the median, + indicates the mean, bounds of box indicate the first and third quartile, and whiskers indicate the min and max. **d, e** Representative images of telomere volumes after treatment with dye (100 nM, 15 min) plus light (660 nm, 10 min) in WT and DDB2 KO cells. **d** U2OS-FAP-TRF1 and **e** RPE-FAP-TRF1 cells. 'n' represents the number of telomeres analyzed for each condition. One-way ANOVA (Sidak multiple comparison test): ***p<0.001, ****p<0.0001. Source data are provided as a Source Data file.
