# Supplementary material for: Global and transcription-coupled repair of 8-oxoG is initiated by nucleotide excision repair proteins
Source: Nat Commun. 2022 Feb 21;13:974. doi: 10.1038/s41467-022-28642-9 (PMC8861037; doi:10.1038/s41467-022-28642-9)
Supplement: Supplementary file 3 — Description of Additional Supplementary Files [file 41467_2022_28642_MOESM3_ESM.docx]

**Description of Additional Supplementary Files**

***Supplementary Movies:***

**Supplementary Movie 1** (Related to Figure 6a, 6b and Supplementary figure 7b, 6d): **DDB2 mediates chromatin decompaction at sites of telomeric 8-oxoG:** Representative movies showing telomere volumes in U2OS WT (Top) and DDB2 KO (Bottom) cells after dye plus light treatment. Cells were fixed 30 minutes post treatment. Telomeres in U2OS WT are bigger than DDB2 KO. Colored scale bar on the left indicates z-axis depth.

**Supplementary Movie 2** (Related to Figure 6c, 6d and Supplementary figure 7c, 7e): **DDB2 mediates chromatin decompaction at sites of telomeric 8-oxoG:** Representative movies showing telomere volumes in RPE WT (Top) and DDB2 KO (Bottom) cells after dye plus light treatment. Cells were fixed 30 minutes post treatment. Telomeres in RPE WT are bigger than DDB2 KO. Colored scale bar on the left indicates z-axis depth.

**Supplementary Movie 3** (Related to Figure 7)**: Working model: role of GG-NER proteins in 8-oxoguanine repair:** DDB2 recognizes 8-oxoG lesions and facilitates chromatin relaxation through chromatin decompaction allowing for recruitment of XPC and OGG1 to the damage site. If DDB2 is retained longer at 8-oxoG sites, the DDB1-Cul4A-RBX1 (CRL) complex mediates auto-polyubiquitylation and degradation of DDB2. After 8-oxoG processing by OGG1, APE1 is recruited to the abasic site generated by OGG1 to facilitate downstream repair.
