# Supplementary material for: Global and transcription-coupled repair of 8-oxoG is initiated by nucleotide excision repair proteins
Source: Nat Commun. 2022 Feb 21;13:974. doi: 10.1038/s41467-022-28642-9 (PMC8861037; doi:10.1038/s41467-022-28642-9)
Supplement: Supplementary file 7 — Reporting Summary [file 41467_2022_28642_MOESM7_ESM.pdf]

nature portfolio

Corresponding author(s): Bennett Van Houten

Last updated by author(s): Jan 21, 2022

Reporting Summary

Nature Portfolio wishes to improve the reproducibility of the work that we publish. This form provides structure for consistency and transparency in reporting. For further information on Nature Portfolio policies, see our Editorial Policies and the Editorial Policy Checklist.

Statistics

For all statistical analyses, confirm that the following items are present in the figure legend, table legend, main text, or Methods section.

n/a	Confirmed	
☐	☒	The exact sample size (*n*) for each experimental group/condition, given as a discrete number and unit of measurement
☐	☒	A statement on whether measurements were taken from distinct samples or whether the same sample was measured repeatedly
☐	☒	The statistical test(s) used AND whether they are one- or two-sided<br>*Only common tests should be described solely by name; describe more complex techniques in the Methods section.*
☐	☒	A description of all covariates tested
☐	☒	A description of any assumptions or corrections, such as tests of normality and adjustment for multiple comparisons
☐	☒	A full description of the statistical parameters including central tendency (e.g. means) or other basic estimates (e.g. regression coefficient) AND variation (e.g. standard deviation) or associated estimates of uncertainty (e.g. confidence intervals)
☐	☒	For null hypothesis testing, the test statistic (e.g. *F*, *t*, *r*) with confidence intervals, effect sizes, degrees of freedom and *P* value noted<br>*Give P values as exact values whenever suitable.*
☒	☐	For Bayesian analysis, information on the choice of priors and Markov chain Monte Carlo settings
☒	☐	For hierarchical and complex designs, identification of the appropriate level for tests and full reporting of outcomes
☒	☐	Estimates of effect sizes (e.g. Cohen's *d*, Pearson's *r*), indicating how they were calculated

Our web collection on statistics for biologists contains articles on many of the points above.

Software and code

Policy information about availability of computer code

Data collection	All data collection described in Methods.<br>1. All immunofluorescence images: Nikon Ti inverted microscope<br>2. Telomere volume measurement: Nikon A1 confocal microscope<br>3. Live-cell imaging: Leica SP5 confocal microscope
Data analysis	Data analysis described in Methods.<br>1. Immunofluorescence was analyzed using NIS elements 5.2.<br>2. Live cell imaging: Leica Application Suite X (LAS X)<br>3. Western blots were analyzed by ImageJ (v1.53k).<br>4. Pulse field gel electrophoresis was analyzed by ImageQuant.<br>5. All other analysis and plotting was done using GraphPad Prism(v8.2).

For manuscripts utilizing custom algorithms or software that are central to the research but not yet described in published literature, software must be made available to editors and reviewers. We strongly encourage code deposition in a community repository (e.g. GitHub). See the Nature Portfolio guidelines for submitting code & software for further information.

Data

Policy information about availability of data

All manuscripts must include a data availability statement. This statement should provide the following information, where applicable:

- Accession codes, unique identifiers, or web links for publicly available datasets
- A description of any restrictions on data availability
- For clinical datasets or third party data, please ensure that the statement adheres to our policy

The data that support the findings of this study are available from the corresponding author upon reasonable request. The source data underlying Figs. 1a-h, 2a-f, 3a-l, 4a-i, 5a-f, 6a-d, and Supplementary Figs. 1a-l, 2a-d, 3a-e, 4a-d, 5a-g, 6a-f, 7a-e are provided as a Source Data file.

Field-specific reporting

Please select the one below that is the best fit for your research. If you are not sure, read the appropriate sections before making your selection.

☒ Life sciences ☐ Behavioural & social sciences ☐ Ecological, evolutionary & environmental sciences

For a reference copy of the document with all sections, see nature.com/documents/nr-reporting-summary-flat.pdf

Life sciences study design

All studies must disclose on these points even when the disclosure is negative.

Sample size	Sample sizes were determined according to commonly accepted standards in the field (Fouquerel et al, PMID:31101499; Jang et al; 31332353). The number of independent experiments and the number of cells scored (n) are indicated in the figures and figure legends.
Data exclusions	No data was excluded.
Replication	At least two to three independent experiments were done for each reported dataset. All results were reproducible.
Randomization	Samples were analyzed in random order. No other randomization was performed.
Blinding	Telomere volume measurements were measured once under blinding conditions, and results were reproduced. Blinding is less feasible for the other assays (such as western blots) and was not performed. All experiments were atleast performed twice independently.

Reporting for specific materials, systems and methods

We require information from authors about some types of materials, experimental systems and methods used in many studies. Here, indicate whether each material, system or method listed is relevant to your study. If you are not sure if a list item applies to your research, read the appropriate section before selecting a response.

Materials & experimental systems

n/a	Involved in the study	
☐	☒	Antibodies
☐	☒	Eukaryotic cell lines
☒	☐	Palaeontology and archaeology
☒	☐	Animals and other organisms
☒	☐	Human research participants
☒	☐	Clinical data
☒	☐	Dual use research of concern

Methods

n/a	Involved in the study	
☒	☐	ChIP-seq
☒	☐	Flow cytometry
☒	☐	MRI-based neuroimaging

Antibodies

Antibodies used	8-oxoG antibody (Trevigen #4354-MC-050), mCherry (1:250; Abcam #ab167453), GFP (1:100, Santa Cruz #B-2), Flag (1:500; CST #14793S), TRF1 (1:500; abcam #10579). Secondary antibodies used: Donkey anti-mouse Alexa488 (1:1000; 1Thermo Fisher Scientific #A21202), Goat anti-Rabbit Alexa-594 (1:1000; Thermo Fisher Scientific #A11012), DDB2 (1:1000; abcam #ab181136), OGG1(1:1000; abcam #124741), Cul4A (1:1000; CST #2699S), DDB1 (1:1000; Invitrogen #37-6200), XPC (1:1000; CST #12701S), CSB (1:1000; abcam #ab96089), mCherry (1:1000; Abcam #ab167453), β-actin (1:30,000; Sigma #A2228), anti-rabbit IgG (1:50,000 Sigma #A0545), anti-mouse IgG (1:50,000 Sigma #A4416), anti-DDB2 (ab181136, Abcam), anti-CUL4A (ab72548, Abcam), anti-CSA (ab137033, Abcam), anti-AQR (A302-547A, Bethyl Laboratories).
Validation	Antibodies were validated by siRNA mediated knockdown as presented in manuscript.

Eukaryotic cell lines

Policy information about cell lines

Cell line source(s)	HEK293T was purchased from ATCC. U2OS-TRF1-FAP and RPE-FAP-TRF1 cell lines were generated in Dr. Patricia Opresko's lab (Fouquerel E et al. Mol Cell. 2019 Jul 11;75(1):117-130; PMID: 31101499). SV40-immortalized MRC-5 cells stably expressing OGG1-GFP or XRCC1-YFP (Menoni H et al. Nucleic Acids Res. 2018 Sep 6;46(15):7747-7756. PMID: 29955842), hTERT-immortalized human fibroblasts VH10 stably expressing GFP-DDB2 (Pines A et al. J Cell Biol. 2012 Oct 15;199(2):235-49; PMID: 23045548), hTERT-immortalized fibroblasts GM01389 (DDB2-deficient; Ouellette MM et al. Hum Mol Genet. 2000 Feb 12;9(3):403-11; PMID: 10655550) stably expressing GFP-DDB2, and XPC-deficient XP4PA-SV expressing XPC-EGFP (Ribeiro-Silva C et al. Nat Commun. 2018 Oct 4;9(1):4067; PMID: 30287812) were obtained from Prof. Dr. Wim Vermeulen.
Authentication	Authentication was performed based on cell morphology and protein expression.
Mycoplasma contamination	All cell lines were tested negative for mycoplasma by MycoAlertTM mycoplasma detection test (Lonza).
Commonly misidentified lines (See ICLAC register)	None of the cells were misidentified on the ICLAC register version 11.
